# Stability of Thawed Apheresis Fresh-Frozen Plasma Stored for up to 120 Hours at 1°C to 6°C

**DOI:** 10.1155/2016/6260792

**Published:** 2016-11-24

**Authors:** William P. Sheffield, Varsha Bhakta, Qi-Long Yi, Craig Jenkins

**Affiliations:** ^1^Centre for Innovation, Canadian Blood Services, Hamilton, ON, Canada; ^2^Department of Epidemiology and Surveillance, Canadian Blood Services, Ottawa, ON, Canada; ^3^Centre for Innovation, Canadian Blood Services, Ottawa, ON, Canada

## Abstract

Regulations concerning the storage of transfusable plasma differ internationally. In Canada, plasma obtained from whole blood donations and frozen within 24 hours of phlebotomy (frozen plasma, FP) may be thawed and transfused within 120 hours of refrigerated storage. However, plasma frozen within 8 hours of phlebotomy following apheresis donation (FFPA) must be transfused within 24 hours of thawing and refrigeration. Our objectives were to measure coagulation factors (F) V, VII, and VIII, fibrinogen activities, and the prothrombin time (PT) in thawed refrigerated FFPA at 0, 24, and 120 hours of storage and to compare these values to those in thawed refrigerated FP. Fibrinogen activity remained unchanged over time, while mean factor levels in 28 FFPA units declined by 17% (FV), 19.7% (FVII), and 54.6% (FVIII) over 120 hours, while PT values rose to 7.6%. Factor activities were significantly higher in FFPA than FP after 120 hours of refrigerated storage. Residual FVIII activities in thawed FFPA met predefined noninferiority criteria compared to thawed FP after 120 hours. These results support a change in Canadian regulations to permit transfusion of thawed FFPA made in a closed system and refrigerated for up to 120 hours, one that could reduce wastage of transfusable plasma.

## 1. Introduction

The ability to freeze transfusable plasma provides both advantages and disadvantages to blood operators and transfusionists. Freezing plasma preserves coagulation factor and other plasma protein activities and makes possible a long shelf life of the frozen product, ranging from one to three years [1]. It also complicates the rapid provision of plasma therapy to patients in urgent need of this intervention because of the time required to thaw frozen plasma. Transfusable plasma is indicated for the prevention or treatment of bleeding due either to genetic deficiencies of coagulation factors for which no purified concentrate is available or to acquired coagulopathy in the setting of disseminated intravascular coagulation, cardiac surgery, warfarin reversal, or massive transfusion [2–8]. It is also indicated as a replacement fluid in plasma exchange for thrombotic thrombocytopenic purpura (TTP), a high volume procedure [9, 10]. European regulations require that plasma be infused as soon as possible after thawing, but in North America, a longer shelf life for thawed, refrigerated plasma is permitted [1].

In the United States and in Canada, the designation “fresh-frozen plasma” (FFP) is applied to plasma that is frozen within 8 hours of phlebotomy. In Canada, transfusable plasma may also be obtained from whole blood donations maintained at 20 to 24°C provided that it is frozen within 24 hours of phlebotomy, yielding frozen plasma (FP). In the United States, two FP-type products may be produced for transfusion: PF24, which is plasma frozen within 24 hours of phlebotomy from whole blood that has been refrigerated within 8 hours of phlebotomy, and PF24RT24, which differs from PF24 only in that the whole blood from which it is derived may be maintained at room temperature for up to 24 hours. FDA guidelines permit FFP, PF24, and PF24RT24 to be thawed and refrigerated for up to 24 hours prior to transfusion; they may all also be relabelled as “Thawed Plasma” and refrigerated for a total of 120 hours (5 days) prior to transfusion, provided that they have been produced in a functionally closed system and are not used in interstate commerce [11]. In large part, the three products are used interchangeably by transfusion medicine practitioners [12, 13]. In Canada, FP may be thawed and refrigerated up to 120 hours prior to transfusion, but thawed FFP must currently be transfused after at most 24 hours of refrigerated storage.

Prior to 2011, Canadian regulators enforced the same 24-hour outdate for thawed refrigerated FP or FFP. We obtained data that allowed for these regulations to be changed by conducting a stability study of refrigerated thawed FP [14]. We demonstrated noninferiority of Canadian FP to PF24 (called FP24 at that time) with respect to coagulation factor VIII (FVIII) activity values in thawed FP refrigerated for 120 hours in an American study by Scott et al. [15], one now cited in FDA guidance documents and a Circular of Information prepared by the AABB, the American Red Cross (ARC), America's Blood Centers (ABC), and the Armed Services Blood Program (ASBP) [11]. We further demonstrated similarity of fibrinogen, FV, and FVII activities and of prothrombin time values to those reported by Scott et al. [14, 15].

We previously elected not to include FFP in our stability studies of thawed plasma. In Canada, excepting the province of Quebec, FFP is currently only available if produced by apheresis (FFPA) [8]. With a view to a potential extension in shelf life of thawed FFPA, in the present study we tested the hypothesis that FVIII activity in thawed refrigerated FFPA was noninferior to FVIII activity in thawed refrigerated FP. We framed the hypothesis in this manner for statistical purposes, and because Canadian regulators accepted this kind of design in our previous study [15] that led to shelf life extension for FP. FVIII is the coagulation factor whose quality control in transfusable plasma is regulated in Canada. Biologically one would expect thermolabile FVIII activity to be better conserved in FFPA due to shorter exposure time of plasma to elevated temperatures. We report data in support of this hypothesis and further show acceptable conservation of fibrinogen, FV, and FVII activities and prothrombin time in thawed refrigerated FFPA.

## 2. Materials and Methods

### 2.1. Design

This study was designed to test the hypothesis that FVIII activity in FFPA would be noninferior to that in FP after both products had been thawed and maintained at 1°–6°C for 120 hours. Because such a shelf life is only considered safe in a transfusable product made in a functionally closed system, we specifically investigated Concurrent Plasma, which is FFPA produced in ~200 mL volumes using a Trima Accel device (Terumo BCT, Lakewood, CO, USA) at the same time as an apheresis platelet product, using Anticoagulant Citrate Dextrose-Formula A (ACD-A) (ACD-FFPA). Sample size was estimated on the basis of the observed FVIII values at 120 hours in our FP stability study [14] and the conservative assumption that the mean ACD-FFPA FVIII values and standard deviations would be identical, which yielded a noninferiority margin of >0.423 IU/mL for *n* = 28. Noninferiority was defined a priori as being confirmed if the lower bound of the 95% confidence interval of the data set was greater than the predetermined noninferiority margin.

Accordingly, 28 ACD-FFPA units produced following standard Canadian Blood Services operating procedures were removed from inventory as an extension of quality control and designated for use in this study. This group comprised 14 non-O type (A, B, or AB) and 14 type O units. Units were tested prior to their one-year expiry date, after having being frozen for between 5 and 11.5 months. For comparisons to FP, we used data from our previous study [14], specifically selecting the results of the 27 units processed using MacoPharma collection sets because these remain in use by the Canadian blood operator and because of the similarity in the size of that data set to the current *n* = 28 study.

### 2.2. Unit Sampling

All ACD-FFPA units were shipped frozen on dry ice from two production sites to the testing site. Units were then stored at −80°C until they were thawed by immersion in a 37°C DH8 (Helmer, Noblesville, IN, USA) quick thaw water bath. Units were then transferred to a Biological Safety Cabinet and mixed gently by brief end-to-end rocking, and aliquots were removed via a sterile sampling coupler using a syringe and a needle. The process was repeated 24 and 120 hours after thaw. At each time point, aliquots were removed and immediately tested, with a backup sample being frozen at −80°C. Units were then returned to the laboratory, where they were stored in a Helmer IB125 refrigerator maintained at 1°–6°C.

### 2.3. Unit Testing

Units were tested in groups of 9 or 10 over a three-week period at the timed intervals described above. All samples were tested on an STA Compact Max automated coagulation analyzer following manufacturer's instructions (Diagnostica Stago, Asnieres, France).

### 2.4. Graphical and Statistical Analysis

Graphical representations of data were produced using GraphPad Prism 6.04 (GraphPad Software, San Diego, CA, USA). Statistical analysis was facilitated using GraphPad InStat or Statistical Package for the Social Sciences (SPSS; IBM, Armonk, NY, USA). Additional details are provided in the text and/or figure legends.

## 3. Results

### 3.1. Noninferiority of Thawed ACD-FFPA after 120 Hours of Refrigerated Storage to FP

After 120 hours of refrigerated storage, the FVIII activity of ACD-FFPA was 0.786 ± 0.23 IU/mL (mean ± SD, *n* = 28). The lower bound of the 95% confidence interval of the data set was calculated to be 0.7124 IU/mL; given that this was greater than the predetermined noninferiority margin of 0.423, noninferiority of stored ACD-FFPA was confirmed with respect to FVIII activity.

### 3.2. Effects of Extended Storage on Coagulation-Related Test Parameters

**Figure 1:**
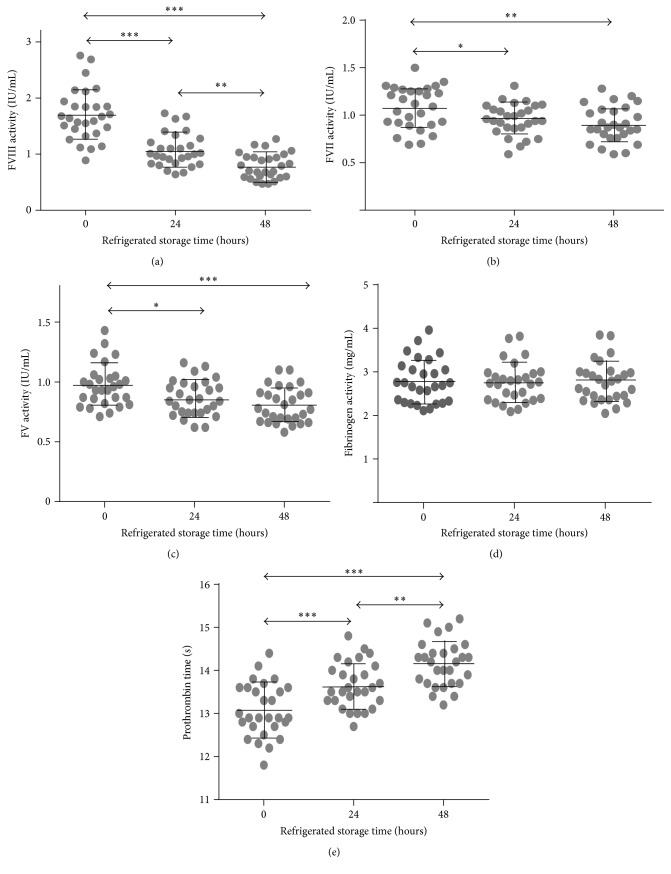
Coagulation parameters in ACD-FFPA. ACD-FFPA units were tested for the activities or times shown on the *y*-axes after thawing (0 hours) and after 24 or 120 hours of refrigerated storage. Grey points represent individual values, while horizontal lines depict the mean and error bars one SD above or below the mean. Lines with arrowheads identify statistical differences among groups by two-way ANOVA with Tukey's post hoc tests: ^*∗*^ *p* < 0.05; ^*∗∗*^ *p* < 0.01; and ^*∗∗∗*^ *p* < 0.001.

As shown in Figure 1 and Table 1, all coagulation factor activities that were tested in ACD-FFPA declined with refrigerated storage time, with the exception of fibrinogen activity, which remained unchanged. The largest decline was observed in FVIII activity, which declined by 38.2% in the first 24 hours and by 54.6% over 120 hours. Part of the magnitude of this decline could be ascribed to the high FVIII activity of ACD-FFPA at thaw (1.73 ± 0.46 IU/mL, compared to our previously determined FP FVIII activity values of 0.901 ± 0.32) [14]. All units in this study had ≥0.70 IU/mL FVIII at thaw, a proportion declining to 96.4% after 24 hours of refrigerated storage and 53.6% after 120 hours.

Reductions in FV and FVII activity followed a similar trajectory, declining on average 11.0 and 11.6%, respectively, in the first 24 hours, and 17.0 and 19.7% after 120 hours. PT values increased by 4.6% in the first 24 hours and by 7.6% after 120 hours.

### 3.3. Comparison to FP Stored under the Same Conditions

**Figure 2:**
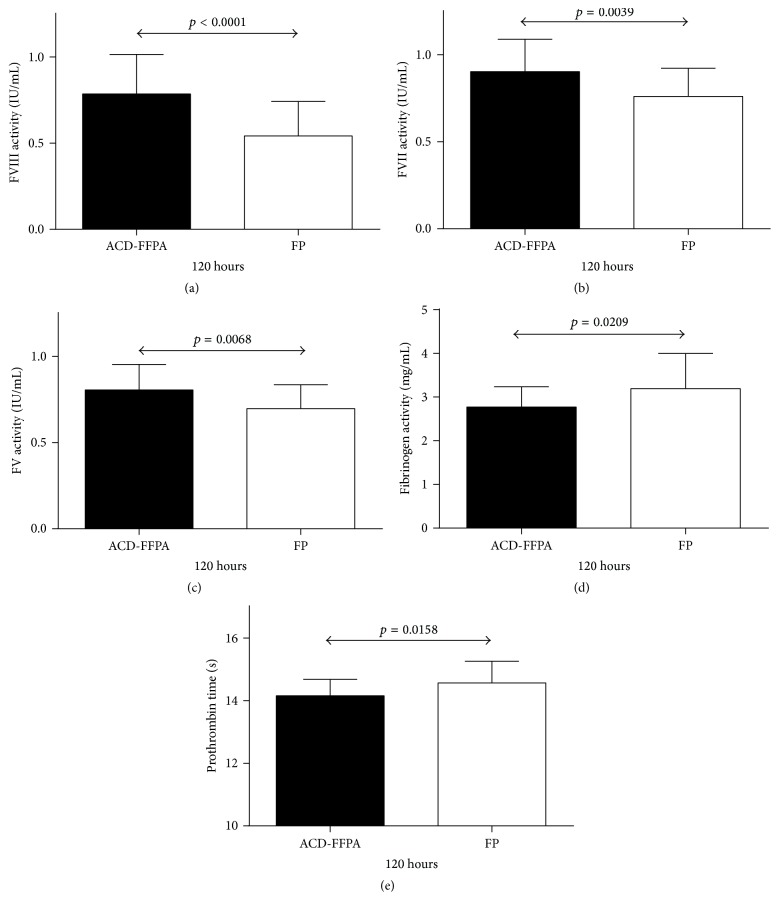
Comparison of coagulation parameters in ACD-FFPA and FP. ACD-FFPA units (solid bars) or FP units (open bars) were tested for the activities or times shown on the *y*-axes after 120 hours of refrigerated storage. Lines with arrowheads identify statistical differences between the two groups, with *p* values above the lines. Data sets (b–e) passing tests of normality and of similarity of standard deviation were tested using the unpaired *t*-test, while Welch's *t*-test was applied to the data in (a).

All parameters tested with ACD-FFPA were compared to previous values for FP following 120 hours of refrigerated storage [14]. As shown in Figure 2 and Table 2, four of the five parameters indicated a greater capacity to support coagulation of ACD-FFPA than FP. FVIII, FVII, and FV activities were significantly higher in ACD-FFPA following extended refrigerated storage. Similarly, mean PT values were also significantly lower, indicative of more rapid clotting of stored ACD-FFPA than stored FP. Only mean fibrinogen activities in stored ACD-FFPA were lower than in stored FP, by approximately 15%.

## 4. Discussion

The primary finding of this study was that ACD-FFPA thawed and maintained at 1°–6°C for 120 hours was noninferior with respect to FVIII activity to FP treated in the same way. Regulators in Canada, the United Kingdom, and Europe, but not the United States, require quality control of transfusable plasma for this labile coagulation factor; in Canada and the United Kingdom, 75% of units tested must contain at least 0.7 IU/mL FVIII activity at thaw [1]. All units tested in this study surpassed this threshold at thaw. As there are no regulations regarding minimum FVIII levels that must be maintained for thawed plasma prior to transfusion, we compared those in ACD-FFPA to those in FP at the end of the storage period. A historical control group published in 2012 was used for the FP data set [14]. Not only were FVIII activity levels significantly higher than those in FP, this finding also held true for another factor considered labile (FV), for a vitamin K-dependent factor (FVII), and for a more global hemostasis test, the PT. Only in fibrinogen activity did stored FP exhibit an apparent minor advantage of 15% greater levels than stored FFPA. It is not clear why fibrinogen activity would be greater in FP than FFPA, but our data set for such values in the current study of ACD-FFPA (with a range of 2.05 to 3.85 g/L) is fully consistent with a study of over 1000 healthy blood donors, 98% of whom were found to exhibit fibrinogen activity levels of 1.8 to 4.2 g/L [16].

Higher levels of FVIII, a labile coagulation factor, are expected in FFP over FP due to the shorter [15] time period between phlebotomy and freezing of the product [17]. Kakaiya et al. reported that plasma from CPDA-1-anticoagulated whole blood donations contained 1.02 ± 0.25 IU FVIII/mL if frozen within 8 hours of phlebotomy, compared to units prepared from refrigerated whole blood donations and frozen within 18–20 hours of phlebotomy, which contained significantly less FVIII activity, 0.55 ± 0.20 IU FVIII/mL; slower processing was associated with reduced FVII but not FV or FXI activities [18]. Similarly Scott et al. reported 0.81 ± 0.19 IU/mL for FFP and 0.66 ± 0.17 IU/mL (*p* < 0.05) for FP24 for FVIII activities at thaw, without significant differences among other clotting factors [15]. We previously demonstrated, at a time when both FFP and FP were made from Canadian whole blood donations, significantly greater FVIII activity in FFP than FP in type O donations (0.89 ± 0.23 IU/mL versus 0.72 ± 0.23 IU/mL) [19].

Our study design allowed us to eliminate trivial explanations for the differences in FVIII activity between ACD-FFPA and FP, such as the known association between ABO blood type and FVIII levels because we compared balanced groups of 14 type O and 14 type A ACD-FFPA units to 14 type O and 13 type non-O FP units [15]. Donors with type O blood have approximately 25% lower levels of FVIII activity, a phenomenon thought to reflect protection by ABO antigens of clearance determinants on von Willebrand Factor, which carries FVIII in the circulation [20].

Although we did not test FFPA or FP for ADAMTS13 in this study or its predecessor, others have demonstrated that this plasma component, of probable importance in plasma exchange in TTP, is present in similar amounts in FFP, FP-type plasma, and cryoprecipitate-poor plasma and is stable in each case to refrigerated storage for 5 days [21]. In addition, we also found ADAMTS13 to be stable in cryosupernatant plasma thawed and refrigerated for 5 days [22].

Only a single previous report in the biomedical literature concerning the quality of ACD-FFPA prepared concurrently to platelets in a functionally closed environment can be found. Sidhu et al. prepared a group of 20 ACD-FFPA units, comprising five units from each ABO blood group [23]. Because these investigators did not sample the units at thaw, but instead at 24, 72, and 120 hours after thaw, a comparison of recoveries between studies is not possible. However, FVIII activities of 0.767 ± 0.048 (mean ± SE) after 120 hours of refrigerated storage were observed, values very similar to those observed in our current study, although it is not clear if the differences in ABO distribution in their sample population had any effect. We elected to use 50% type O and 50% nontype O in our group of analyzed units to more closely mimic our donor population in Canada (46% type O), whereas Sidhu et al. used a 25% type O and a 75% nontype O mixture of units [20]. Sidhu et al. also found no change in fibrinogen or FVII levels between Days 1 and 5 of refrigerated storage and a statistically significant loss of FV activity, mirroring our results [23]. Similarly, Neisser-Svae et al. found residual FVIII activities in thawed FFP of 0.75 ± 0.13 IU/mL (mean ± SD) after 5 days of refrigerated storage [24], and von Heymann et al. reported median FVIII values of 0.75 with an interquartile (25–75%) range of 0.68 to 0.88 IU/mL for thawed FFP, again showing strong similarity to our results with ACD-FFPA [25].

In our previous stability studies of thawed FP and thawed cryosupernatant plasma, at the conclusion of the studies, we tested the residual products in the bag for bacterial contamination [14, 22]. A total of 90 units were negative for growth in the BacT-ALERT system employed in Canada for mandatory screening of platelet products. Since bacterial contamination of plasma is a very rare event, these findings suggested that our procedures were sufficiently robust to avoid contamination of plasma units by airborne microbes despite multiple sampling events. For this reason BacT-ALERT testing was not performed in the current study.

One reason that we did not study FFPA stability when we previously addressed the issue of thawed FP stability was that some FFPA in the Canadian blood operator's inventory is generated in a functionally open system, necessitating transfusion within four hours of thawing, and some (ACD-FFPA) is not. However, in the interval since that initial study, the trend towards the maintenance of trauma packs containing prethawed FP units by Canadian trauma centres for use in massive transfusion has intensified [26]. Hospitals cannot currently follow plasma-sparing practices, such as releasing thawed trauma pack FP for transfusion to nontrauma patients on Day 4 of refrigerated storage with FFPA due to its current short regulated shelf life. Concerns over potential confusion between FFPA from functionally open and closed systems have also dissipated, as Concurrent Plasma, ACD-FFPA, is now uniquely identifiable in the Canadian Blood Services inventory due to not only its anticoagulant, but also more importantly due to its being the only FFP distributed to Canadian hospitals in ~200 mL volumes. The results of this study provide support to changing the permitted refrigerated shelf life of ACD-FFPA to 120 hours, aligning Canadian practice to that in the United States, where transfusion of thawed relabeled FFP or PF24 stored refrigerated for up to 120 hours has been permitted since 1998, with no apparent negative consequences for patients [12, 13].

## Figures and Tables

**Table 1:** Stability of coagulation parameters in ACD-FFPA.

TEST	Time of refrigerated storage (hours)		
	0	24	120
FV (IU/mL)	0.971 ± 0.18	0.864 ± 0.15 (89)^*∗*^	0.806 ± 0.15 (83)^*∗∗∗*^
FVII (IU/mL)	1.08 ± 0.22	0.955 ± 0.19 (88)^*∗*^	0.904 ± 0.16 (84)^*∗∗∗*^
FVIII (IU/mL)	1.73 ± 0.46	1.07 ± 0.30 (62)^*∗∗∗*^	0.786 ± 0.23 (45)^*∗∗∗*^
Fibrinogen (grams/L)	2.79 ± 0.50	2.76 ± 0.45 (99)	2.77 ± 0.47 (99)
PT (seconds)	13.1 ± 0.62	13.7 ± 0.53 (105)^*∗∗∗*^	14.1 ± 0.53 (108)^*∗∗∗*^

Values are reported as the mean ± one standard deviation; ^*∗*^ *p* < 0.05 and ^*∗∗∗*^ *p* < 0.001 versus 0 hours value for each parameter. Parenthetical values are the activity or clotting time at time *t* as a percentage of that at time *t* = 0.

**Table 2:** Comparison of coagulation parameters in ACD-FFPA and FP^a^ at 120 h.

TEST	ACD-FFPA^b^	FP^c^	*p* value
FV (IU/mL)	0.806 ± 0.15	0.697 ± 0.14	0.0068
FVII (IU/mL)	0.904 ± 0.19	0.761 ± 0.16	0.0039
FVIII (IU/mL)	0.786 ± 0.23	0.542 ± 0.20	<0.0001
Fibrinogen (grams/L)	2.77 ± 0.47	3.193 ± 0.81	0.0209
PT (seconds)	14.1 ± 0.53	14.6 ± 0.69	0.0158

^a^Values are reported as the mean ± one standard deviation. Statistical comparisons were made using an unpaired *t*-test in all cases, except for FVIII, for which the unpaired *t*-test, Welch corrected, was employed. ^b^From current study. ^c^From [11].
